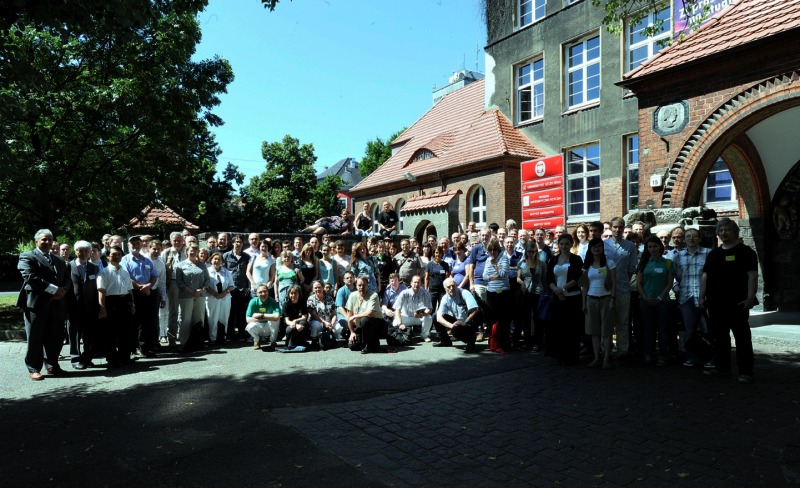# Introduction to the Special Collection of Papers from EANA 2013: The 13th European Workshop on Astrobiology (“Through Cosmic Dust to DNA”): 22-25 July 2013, Szczecin, Poland

**DOI:** 10.1007/s11084-014-9364-7

**Published:** 2014-10-28

**Authors:** Franco Ferrari, Ewa Szuszkiewicz

**Affiliations:** CASA* and Institute of Physics, University of Szczecin, ul. Wielkopolska 15, 70-451 Szczecin, Poland

EANA 2013 took place from the 22nd to the 25th July 2013 in the Faculty of Mathematics and Physics of the University of Szczecin. It was the 13th workshop organized by EANA (European Astrobiology Network Association), an annual event which is hosted in one of the member countries and is recognized to be the most important happening in the astrobiological calendar in Europe.

EANA (web page: www.astrobiology.pl/eana) started its activity in 2001 with the aim of bringing together European researchers interested in astrobiology programmes and stimulating their collaboration across the borders of different scientific disciplines. Particular attention is directed to introducing young scientists to this interdisciplinary field of studies and to disseminating the results of astrobiological research to its relevant beneficiaries as well as to students and the general public. EANA is a network of 19 European nations active in astrobiology: Austria, Belgium, Czech Republic, Denmark, Finland, France, Germany, Greece, Hungary, Italy, Poland, Portugal, Romania, Russia, Spain, Sweden, Switzerland, The Netherlands and United Kingdom. Astrobiology groups in Brazil, China, Japan, Mexico and USA are associated members.

The inauguration of EANA was announced during the First European Workshop on Astrobiology co-organized together with European Space Agency (ESA) at the ESRIN (European Space Research Institute) in Frascati (Italy) in spring 2001. Since then the astrobiology community in Europe has steadily increased year after year and the regular succession of EANA’s workshops has measured the pace of the research developments in astrobiology. The previous editions of the European Workshop on Astrobiology have been held in the following places:

EANA 2001: May 21–23, 2001, Frascati, Italy

EANA 2002: September 16−19, 2002, Graz, Austria

EANA 2003: November 18–20, 2003, Madrid, Spain

EANA 2004: November 22–25, 2004, Milton Keynes, United Kingdom

EANA 2005: October 10–12, 2005, Budapest, Hungary

EANA 2006: October 16–18, 2006, Lyon, France

EANA 2007: October 22–24, 2007, Turku, Finland

EANA 2008: September 1–3, 2008, Neuchâtel, Switzerland

EANA 2009: October 12–14, 2009, Brussels, Belgium

EANA 2010: September 6–8, 2010, Pushchino, Russia

EANA 2011: July 11–14, 2011, Köln, Germany

EANA 2012: October 15–17, 2012, Stockholm, Sweden

The next Workshop will take place in Edinburgh, United Kingdom, October 13–16, 2014.

Poland is an EANA member since 2003. On the occasion of the 10th anniversary of Poland’s presence in EANA, the annual EANA workshop was hosted in this country. The Polish astrobiology is represented by CASA* (Centre for Advanced Studies in Astrobiology and Related Topics), which since 2007 is formally organized as a consortium of five Founding Institutions: the Nicolaus Copernicus Astronomical Center (CAMK) of the Polish Academy of Science (PAS) in Warsaw, the Space Research Center (CBK) of PAS in Warsaw, the Institute of Paleobiology of PAS in Warsaw, the Nicolaus Copernicus University in Toruń and the University of Szczecin. The Headquarters of CASA* is the Faculty of Mathematics and Physics of the University of Szczecin. The main goals of the Centre are to stimulate, perform and coordinate interdisciplinary research in astrobiology in Poland; to develop advanced technologies and to promote their commercial exploitation; to promote the collaboration on astrobiological topics of Polish research teams with other countries in Europe; to train the next generation of astrobiology researchers and to increase the public awareness for science. EANA 2013 has been a very good opportunity to promote astrobiology in Poland.

The 13th EANA Workshop on Astrobiology (web page: http://eana13.astrobiologia.pl/) was held in the building of the Faculty of Mathematics and Physics of the University of Szczecin near to the beautiful Kasprowicz Park and the historical City Hall. The list of main topics of the meeting is provided below:

- Astrochemistry, interstellar medium;
- Astrophysics, protoplanetary discs and planets;
- Planetary habitability and exploration;
- Macromolecules and models of prebiotic molecules;
- Origin and evolution of life, extremophiles;
- Rocks, fossils and meteorites;
- Space technology, medicine and industry;
- Miscellaneous subjects in astrobiology.

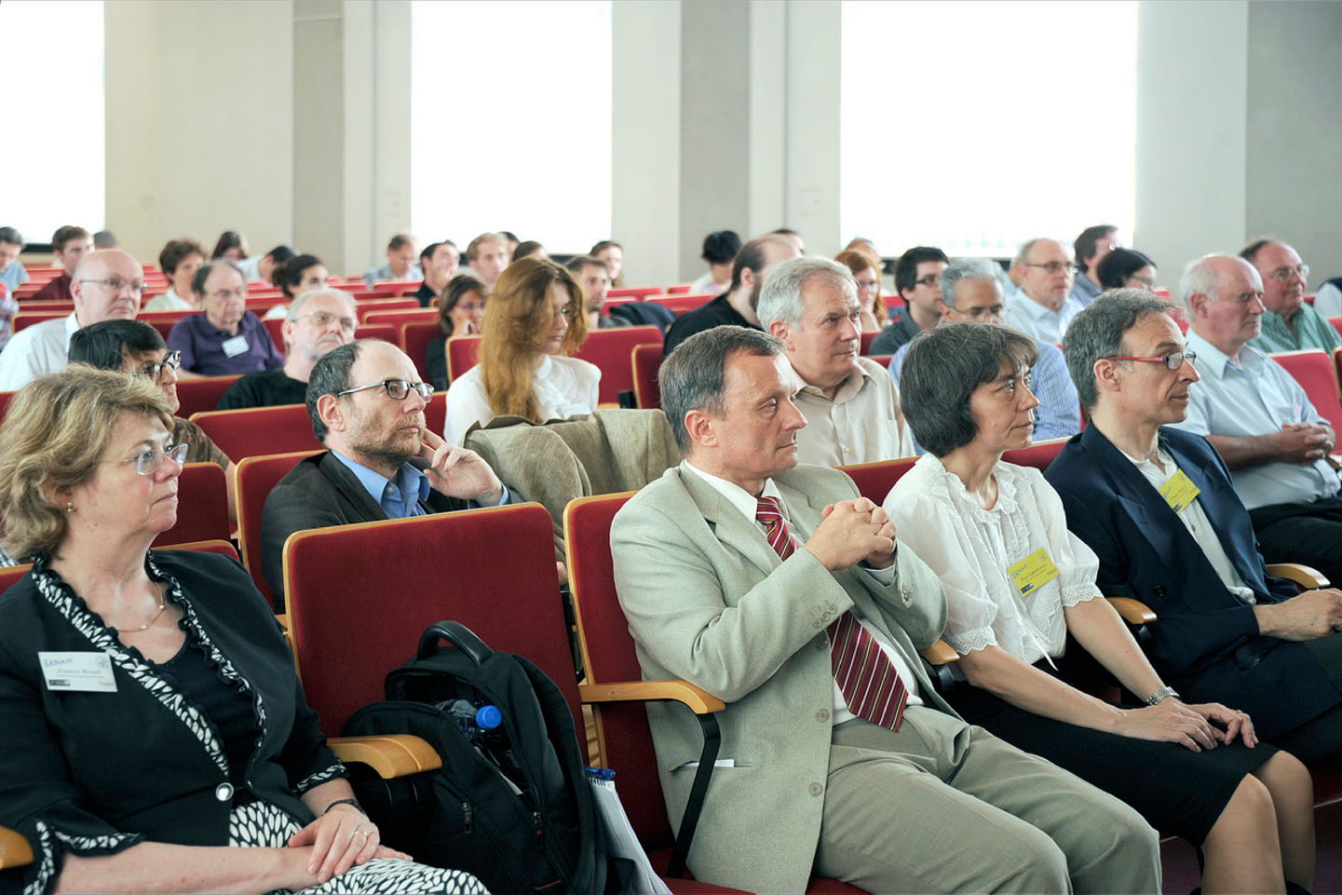


Picture of the participants during the opening session of the workshop which took place on Monday, 22nd of July 2014 in the main lecture hall of the Faculty of Mathematics and Physics, University of Szczecin (in the first row from left to right: the newly elected President of EANA – Frances Westall, the Vice-Rector for Science and International Cooperation of the University of Szczecin – Marek Górski, the coordinators of CASA* and main organisers of EANA 2013 – Ewa Szuszkiewicz and Franco Ferrari). [image courtesy of Jerzy Giedrys]

The Scientific Organising Committee was composed by the following scientists: André Brack, CNRS (France), Axel Brandenburg, NORDITA (Sweden), Charles Cockell, University of Edinburgh (UK), Pascale Ehrenfreund, SPI (USA), Franco Ferrari, CASA*, University of Szczecin (Poland), David Field, Aarhus University (Denmark), Beda Hofmann, Natural History Museum, Bern (Switzerland), Gerda Horneck, DLR Cologne (Germany), Natalia B. Gontareva, St.Petersburg State Polytechnical University (Russia), Zbigniew Kłos, SRC, PAS, Warsaw (Poland), Józef Kaźmierczak, IP, PAS, Warsaw (Poland), Kirsi Lehto, University of Turku (Finland), Jacek Krełowski, NCU Toruń (Poland), Helmut Lammer, IWF (Austria), Nigel Mason, Open University (UK), Christian Muller, B.USOC (Belgium), Juan Pérez-Mercader, Origins of Life Initiative, Harvard University (USA), Petra Rettberg, DLR Cologne (Germany), Heike Rauer, TU Berlin and DLR Berlin (Germany), Györgyi Rontó, Hungarian Academy of Sciences, Budapest (Hungary), Alessandra Rotundi, University of Naples “Parthenope” (Italy), François Raulin, University of Paris 12 and 7 (France), Helga Stan-Lotter, University of Salzburg (Austria), Ewa Szuszkiewicz CASA*, University of Szczecin (Poland), Jorge Vago, ESA (The Netherlands), Frances Westall, CNRS, Orléans (France).

To EANA 2013 gathered 118 participants coming from 25 countries (the 19 European countries already mentioned above, Brazil, Chile, Iran, Ireland, Japan and USA). During 10 scientific sessions as many as 49 talks have been delivered, including 12 keynote lectures. Every evening was dedicated to poster viewing. In total 50 posters were presented. Following the tradition, a student contest was organized for the best students’ oral and poster contributions. This year the prize was given to Jan Frösler from the University of Duisburg-Essen in Germany. Furthermore, awards were made to the eight best posters. During EANA 2013 also a special prize of CASA* for the best talks of young scientists has been assigned to René Heller (Leibniz-Institute for Astrophysics in Potsdam, Germany, currently at the McMaster University, Hamilton, Canada) and Rafał Wieczorek (Center for Fundamental Living Technology, University of Southern Denmark in Odense, Denmark). The scientific contributions to this conference have been collected in this Issue of “Origin of Life and Evolution of Biospheres”. We are very grateful to Alan Schwartz for making this happen.

Several interesting events have been associated with EANA 2013, namely the Public Lecture “Our cosmic genealogy” given by the outstanding Polish astrophysicist prof. Michał Różyczka from CAMK, the presentation of BRITE-PL - the first Polish Science Satellite performed by the Project Manager Tomasz Zawistowski from CBK and the field trip to the The “Morasko Meteorite” Reserve. The conference dinner included some elements of the molecular cuisine.

The Local Organizing Committee would like to thank for support EANA, ESA, the Polish Academy of Sciences, the Polish Ministry of Science and Higher Education, the Polish Astronomical Society, the Committee for Space Research, the Springer publisher, the University of Szczecin, the Faculty of Mathematics and Physics of the University of Szczecin and CASA*. Partial support from the Marshal of the Westpomeranian Voivodeship is also gratefully acknowledged.
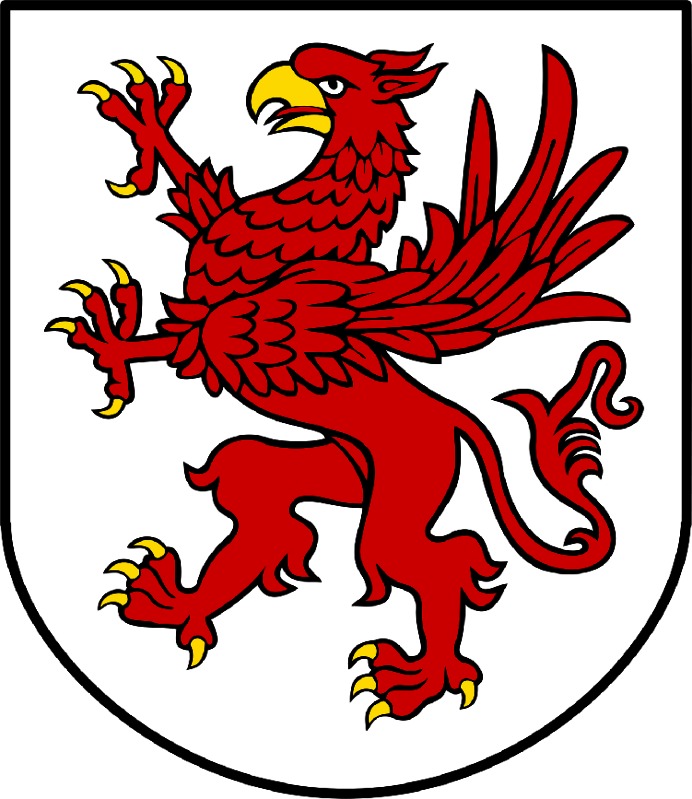


Picture of the participants in EANA 2013 made in front of the conference venue, the building of the Faculty of Mathematics and Physics of the University of Szczecin. [image courtesy of Jerzy Giedrys].